# Supplementary material for: Influence of Soil-Borne Inoculum of Plasmodiophora brassicae Measured by qPCR on Disease Severity of Clubroot-Resistant Cultivars of Winter Oilseed Rape (Brassica napus L.)
Source: Pathogens. 2021 Apr 6;10(4):433. doi: 10.3390/pathogens10040433 (PMC8067420; doi:10.3390/pathogens10040433)
Supplement: Supplementary file 1 [file pathogens-10-00433-s001.zip › pathogens-1167562-supple/pathogens-1167562-supplementary-figure.docx]

(a)

(b)

**Figure. S1.** Daily precipitation (mm) and mean air temperature (ºC) at Borrby weather station (55.4°N; 14.2°E) in southern Sweden and at Kumla weather station (59.2°; 15.3°) in central Sweden from August 1 to October 10: (a) 2017 and (b) 2018.
