# Supplementary material for: Influence of Soil-Borne Inoculum of Plasmodiophora brassicae Measured by qPCR on Disease Severity of Clubroot-Resistant Cultivars of Winter Oilseed Rape (Brassica napus L.)
Source: Pathogens. 2021 Apr 6;10(4):433. doi: 10.3390/pathogens10040433 (PMC8067420; doi:10.3390/pathogens10040433)
Supplement: Supplementary file 1 [file pathogens-10-00433-s001.zip › pathogens-1167562-supple/pathogens-1167562-supplementary-tables.docx]

Table S1. Seed yield (9% water content (wc)), oil yield (of dry matter (DM)), oil content at 9% wc at harvest and plant density in the four winter oil seed rape field trials harvested 23–31 July 2018 for Cultivar mix (S); clubroot resistant cultivars (CR) cv. Mentor, cv. Alister and cv. Archimedes. Selection of field sites according to field tests for abundance of gene copies g^-1^ soil of *Plasmodiophora brassicae* DNA; at Simrishamn 1 100 000; Tomelilla 2 500 000; at Kumla 15 000; at Hallsberg 2 500 analysed immediately before seeding

|  | | **Seed yield** | | **Relative**  **yield** | **Oil yield** | | **Oil content** | | **Plant density autumn** | **Plant density spring** |
| --- | --- | --- | --- | --- | --- | --- | --- | --- | --- | --- |
| **Treatment** | | **(kg ha^-1^)** | |  | **(kg DM ha^-1^)** | | **(% at 9% wc)** | | **(No. m^-2^)** | **(No. m^-2^)** |
| *Simrishamn^1^* | |  |  |  |  |  |  |  |  |  |
|  | ‘Cultivar mix‘ | 2 359 | b* | 100 | 1 555 | a | 53,3 | a | 44 | 32 |
|  | cv. Mentor | 3 197 | a | 136 | 1 511 | ab | 53,5 | a | 56 | 42 |
|  | cv. Alister | 3 219 | a | 136 | 1 474 | ab | 51,5 | b | 51 | 38 |
|  | cv. Archimedes | 3 116 | a | 132 | 1 145 | b | 52,0 | b | 49 | 46 |
|  | *p* | 0.015 | |  | 0.026 | | <0.001 | | ns | ns |
|  | CV | 12.1 | |  | 12.5 | | 0.8 | | 18.3 | 18.7 |
| *Tomelilla^2^* | |  |  |  |  |  |  |  |  |  |
|  | ‘Cultivar mix‘ | 3 026 | b | 100 | 1 454 | b | 52,7 | a | 55 | 36 |
|  | cv. Mentor | 4 634 | a | 153 | 2 226 | a | 52,8 | a | 49 | 42 |
|  | cv. Alister | 4 779 | a | 158 | 2 204 | a | 50,7 | b | 56 | 51 |
|  | cv. Archimedes | 4 631 | a | 153 | 2 145 | a | 50,9 | b | 48 | 45 |
|  | *p* | <0.001 | |  | <0.001 | | <0.001 | | ns | ns |
|  | CV | 8.7 | |  | 9.3 | | 0.6 | | 28.1 | 34.2 |
| *Kumla^3^* | |  |  |  |  |  |  |  |  |  |
|  | ‘Cultivar mix‘ | 2 548 | b | 100 | 1 143 | b | 49,3 | ab | 44 | 34 |
|  | cv. Mentor | 2 871 | ab | 113 | 1 218 | ab | 46,6 | c | 43 | 35 |
|  | cv. Alister | 2 950 | a | 116 | 1 331 | a | 49,6 | a | 41 | 33 |
|  | cv. Archimedes | 2 569 | ab | 101 | 1 096 | ab | 46,9 | bc | 40 | 34 |
|  | *p* | 0.017 | |  | 0.011 | | 0.007 | | ns | ns |
|  | CV | 6.7 | |  | 7.1 | | 2.6 | | 11.1 | 25.8 |
| *Hallsberg^4^* | |  |  |  |  |  |  |  |  |  |
|  | ‘Cultivar mix‘ | 4 310 | a | 100 | 1 984 | a | 50,6 | a | 42 | 37 |
|  | cv. Mentor | 3 873 | b | 90 | 1 724 | c | 48,9 | b | 42 | 39 |
|  | cv. Alister | 4 052 | b | 94 | 1 860 | b | 50,5 | a | 45 | 39 |
|  | cv. Archimedes | 3 980 | b | 92 | 1 771 | bc | 48,9 | b | 42 | 34 |
|  | *p* | <0.001 | |  | <0.001 | | <0.001 | | ns | ns |
|  | CV | 2.7 | |  | 2.7 | | 0.9 | | 8.6 | 8.8 |

Seeding date: ^1^25 August; ^2^26 August; ^3^9 August; ^4^15 August 2017. * Different letters indicate significant differences according to Tukey’s HSD-test (*p<0.05*).

Table S2. Seed yield (9% water content (wc)) and oil yield (of dry matter (DM)), oil content at 9% wc, protein content and chlorophyll content in the harvested seed, which correspond to maturity level in winter oil seed rape at harvest at three winter oil seed rape field trials harvested on 30 July-5 Aug 2019 for the clubroot resistant cultivars cv. Mentor, cv. Alister, cv. Archimedes and a susceptible Cultivar mix. The field trial sites were selected according to field tests measuring the abundance of gene copies per gram^-1^ soil of *Plasmodiophora brassicae* DNA; at Simrishamn 600 000; Tomelilla 370 000; at Kumla 50 000

|  | | **Seed yield** | | **Relative**  **yield** | **Oil yield** | | **1000-seed weight** | | **Oil content** | | **Protein** | | **Plant density autumn** | | **Plant density spring** | | **Chlorophyll content** | |
| --- | --- | --- | --- | --- | --- | --- | --- | --- | --- | --- | --- | --- | --- | --- | --- | --- | --- | --- |
| **Treatment** | | **(kg ha^-1^)** | |  | **(kg DM ha^-1^)** | | **(g at 15% wc)** | | **(% at 9% wc)** | | **(% of DM)** | | **(No. m^-2^)** | | **(No. m^-2^)** | | **(ppm)** | |
| *Simrishamn*^1^ | |  |  |  |  |  |  |  |  |  |  |  |  |  |  |  |  |  |
|  | ‘Cultivar mix‘ | 4 201 | a* | 100 | 1 798 | a | 4.32 | b | 43.9 | a | 20.1 | b | 55 |  | 53 |  | 14.9 | b |
|  | cv. Mentor | 4 207 | a | 100 | 1 791 | ab | 4.78 | a | 44.5 | a | 20.5 | b | 43 |  | 50 |  | 11.9 | b |
|  | cv. Alister | 3 305 | b | 79 | 1 422 | b | 4.65 | a | 41.3 | b | 20.5 | b | 51 |  | 49 |  | 19.4 | a |
|  | cv. Archimedes | 3 773 | ab | 90 | 1 620 | ab | 4.29 | b | 41.5 | b | 21.8 | a | 65 |  | 53 |  | 21.9 | a |
|  | *p* | 0.004 | |  | 0.037 | | <0.001 | | <0.001 | | <0.001 | | ns | | ns | | <0.001 | |
|  | CV | 7.9 | |  | 10.8 | | 2.8 | | 107 | | 1.8 | | 48.0 | | 46.7 | | 76.3 | |
| *Tomelilla*^2^ | |  |  |  |  |  |  |  |  |  |  |  |  |  |  |  |  |  |
|  | ‘Cultivar mix‘ | 5 462 |  | 100 | 2 500 |  | 4.62 | b | 45.8 | a | 18.2 | b | 62 |  | 63 |  | 11.9 |  |
|  | cv. Mentor | 5 247 |  | 96 | 2 430 |  | 5.16 | a | 46.3 | a | 18.5 | b | 52 |  | 51 |  | 12.3 |  |
|  | cv. Alister | 5 525 |  | 101 | 2 442 |  | 5.29 | a | 44.2 | b | 18.3 | b | 56 |  | 56 |  | 13.5 |  |
|  | cv. Archimedes | 5 686 |  | 104 | 2 520 |  | 4.78 | b | 44.4 | b | 19.6 | a | 55 |  | 53 |  | 11.2 |  |
|  | *p* | ns | |  | ns | | <0.001 | | <0.001 | | 0.008 | | ns | | ns | | ns | |
|  | CV | 4.4 | |  | 4.7 | | 1.7 | | 122 | | 2.8 | | 13.1 | | 17.3 | | 72.5 | |
| *Kumla*^3^ | |  |  |  |  |  |  |  |  |  |  |  |  |  |  |  |  |  |
|  | ‘Cultivar mix‘ | 4 017 | a | 100 | 1 902 | a | 5.69 |  | 47.3 | a | 18.4 | b | 46 |  | 34 | a | 10.2 | b |
|  | cv. Mentor | 4 589 | a | 114 | 2 157 | a | 5.60 |  | 47.0 | a | 18.0 | b | 45 |  | 40 | a | 11.1 | b |
|  | cv. Alister | 4 260 | a | 106 | 1 953 | a | 5.95 |  | 45.8 | ab | 18.2 | b | 45 |  | 35 | a | 10.3 | b |
|  | cv. Archimedes | 2 893 | b | 72 | 1 285 | b | 5.77 |  | 44.3 | b | 20.5 | a | 48 |  | 21 | b | 14.9 | a |
|  | *p* | <0.001 | |  | <0.001 | | ns | | 0.013 | | 0.007 | | ns | | <0.001 | | 0.007 | |
|  | CV | 9.9 | |  | 11.3 | | 4.3 | | 3.7 | | 4.8 | | 10.1 | | 14.4 | | 4.1 | |

Seeding date: ^1^17 August; ^2^21 August; ^3^15 August 2018.

* Different letters indicate significant differences according to Tukey’s HSD-test (*p<0.05*)

Tabell S3. Locations and soil parameters at soil depth 0-30 cm, including the number of gene copies of *Plasmodiophora brassicae*, prior to seeding in the original soil for field trials of testing cultivars of resistant winter oil seed rape seeded in 2017 and in 2018

| **Location** | **Coordinates** | **Clay content (%)** | **Soil pH** | **Soil org. matter**  **(%)** | **P-AL**  **mg 100 g^-1^** | **K-AL**  **mg 100 g^-1^** | **Ca-AL**  **mg 100 g^-1^** | **Gene copies of *P. brassicae***  **g^-1^ soil*** |
| --- | --- | --- | --- | --- | --- | --- | --- | --- |
| 2017 |  |  |  |  |  |  |  |  |
| Simrishamn | N ´55.5º  E ´14.3º | 14 | 6.5, 7.0, 6.8^a^ | 2.7 | 13 | 12 | 170 | 1 100 000 |
| Tomelilla | N ´55.5º  E ´14.0º | 14 | 6.5, 6.6, 7.1 | 3.1 | 5.2 | 15 | 160 | 2 500 000 |
| Kumla | N ´59.6º  E ´15.5º | 16 | 6.1, 6.6, 6.9 | 1.9 | 13 | 9.7 | 146 | 15 000 |
| Hallsberg | N ´59.2º  E ´14.6º | 11 | 6.4, 6.1, 7.0 | 7.2 | 10 | 19.8 | 293 | 2 500 |
| 2018 |  |  |  |  |  |  |  |  |
| Simrishamn | N ´55.5º  E ´14.3º | 18 | 6.3, 6.3, 6.7 | 3.4 | 8.6 | 16 | 180 | 600 000 |
| Tomelilla | N ´55.5º  E ´14.0º | 17 | 6.9, 7.0, 7.4 | 2.5 | 6.8 | 6.6 | 180 | 370 000 |
| Kumla | N ´59.6º  E ´15.5º | 22 | 6.0, 6.3, 6.8 | 3.4 | 5.8 | 14.7 | 197 | 15 000 |

* Field analysis taken prior to choosing the field experimental sites. ^a^ pH at soil depth 0-30 cm, 30-60 and 60-90 cm, respectively.
